# Social Media Use for Public Health Campaigning in a Low Resource Setting: The Case of Waterpipe Tobacco Smoking

**DOI:** 10.1155/2015/562586

**Published:** 2015-07-26

**Authors:** Mohammed Jawad, Jooman Abass, Ahmad Hariri, Elie A. Akl

**Affiliations:** ^1^Department of Primary Care and Public Health, Imperial College London, London W6 8RP, UK; ^2^Poole, UK; ^3^Faculty of Medicine, Imperial College London, London W6 8RP, UK; ^4^Department of Internal Medicine, American University of Beirut, Beirut 1107 2020, Lebanon; ^5^Department of Medicine, State University of New York at Buffalo, New York 14214, USA; ^6^Department of Clinical Epidemiology and Biostatistics, McMaster University, Hamilton, Canada L8S 4L8

## Abstract

*Introduction*. Waterpipe tobacco smoking prevalence is increasing worldwide despite its documented health effects. A general belief that it is less harmful than cigarettes may be fuelled by the lack of media campaigns highlighting its health effects. We aimed to create and assess the impact of a social media campaign about dangers of waterpipe smoking. *Methods*. The “ShishAware” campaign included three social media (Facebook, Twitter, and YouTube) and a website. Nine months after launch we collected data to assess use of, and reaction to, our media accounts. *Results*. Requiring limited maintenance resources, Facebook attracted campaign supporters but YouTube attracted opposers. Twitter enabled the most organisation-based contact but Facebook was the most interactive medium. Facebook users were more likely to “like” weekday than weekend statuses and more likely to comment on “shisha fact” than “current affairs” statuses. Follower subscription increased as our posting rate increased. Our YouTube video gained 19,428 views (from all world continents) and 218 comments (86% from pro-waterpipe smokers). *Conclusions*. Social media campaigns can be created and maintained relatively easily. They are innovative and have the potential for wide and rapid diffusion, especially towards target audiences. There is a need for more rigorous evaluation of their effects, particularly among the youth.

## 1. Introduction

Tobacco smoking using waterpipe—also known as nargileh, hookah, and shisha—is traditional to the Middle Eastern and South Asian region although its prevalence is alarmingly high among school students and university students in the Middle East and among groups of Middle Eastern descent in Western countries [1]. Among high school students in London, the prevalence of waterpipe tobacco smoking was over double that of cigarette smoking (7.6% versus 3.4%) [2], whereas in the US national reports suggest 2.6% of adolescents are current waterpipe users [3].

Studies have found that waterpipe tobacco smoking is significantly associated with lung cancer, respiratory illness, low birth weight, and periodontal disease [4, 5]. There are also possible associations with bladder cancer, nasopharyngeal cancer, oesophageal cancer, oral dysplasia, and infertility [4], which are expected in lieu of the high level of toxicants found in waterpipe tobacco smoke aerosol [6]. In spite of both the proven and suspected deleterious health effects, waterpipe users widely believe it to be less harmful and a safer alternative to cigarette smoking [7, 8]. They believe it contains less nicotine, that the water has filtering properties, and that switching from cigarettes to waterpipe would reduce their health risks [9–11]. In one study, respondents considered that the lack of media campaigns implies that waterpipe smoking must be safer than cigarette smoking [10].

Social media is a popular method of communication and a regular source of information for internet users, including health information [12]. It has been shown to reach wide audiences; however its position in public health campaigning is relatively new. Importantly, it can be used as a powerful tool for promotion of the tobacco industry [13] yet we remain unsure how best to use social media campaigns for the purposes of prevention [14].

In order to address this emergent public health problem, we have conducted a public health campaign, entitled “ShishAware,” to raise awareness about the health risks of waterpipe tobacco smoking. The objective of this paper is to describe the use of social media in conducting this campaign, for tobacco control purposes.

## 2. Materials and Methods

### 2.1. Campaign Description

The ShishAware campaign was an unfunded, grassroots initiative which aimed to raise awareness about the health risks of waterpipe tobacco smoking. While the campaign included a number of field activities, it mainly relied on three social media (Facebook, Twitter, and YouTube) and a campaign website. The target audience of the campaign was the local government organisations (through the field activities) but also the wider global community (through the social media). The campaign had no formal funding.

### 2.2. Campaign Team

The campaign team consisted of two fifth year medical students (Mohammed Jawad and Ahmad Hariri) and a pharmacist (Jooman Abass). The team members initiated the campaign and worked on it on a volunteer basis. They did not have any formal training in using social media but were experienced through their personal use.

Accurate and relevant health information was sought by conducting a literature review on waterpipe tobacco smoking, prioritising systematic reviews and peer-reviewed papers. These were synthesised into “shisha facts”—short, snappy pieces of information, for example, “shisha is just as bad for your lung function as cigarettes (Raad et al., 2011)” [5]. We also posted current affairs information by using the online content retriever “Google Alerts,” a service which automatically scans for new online news articles based on specific key words [15], and these were screened for newsworthiness.

### 2.3. Development of Social Media

Facebook and Twitter accounts were created in less than an hour, and ShishAware posted “shisha facts” and global news articles, as well as general communication posts with its audience. ShishAware was advertised by posting content on other, related social media accounts. It caught the attention of a local government in London, who collaborated on a waterpipe tobacco smoking awareness video for young people and invited ShishAware to speak at meetings, youth workshops and conduct lesson plans for fifteen local high schools. The video was designed by young people and its content included background information on waterpipe smoking, excerpts from interviews with (i) young people who had just attended a workshop on waterpipe smoking, (ii) an ex-waterpipe smoker, who detailed reasons for cessation, (iii) one of the ShishAware members, who explained the aims of this campaign, and (iv) general public. It ended with information on the health effects of waterpipe smoking and maintained an “It's your choice” message throughout. The video can be found at https://www.youtube.com/watch?v=sWTgzJGzGv4. No methods were used to promote the waterpipe tobacco smoking awareness video. The website was created using WordPress, a user-friendly, free website-building program with the help of other team members, and included evidence-based information.

### 2.4. Evaluation of the Campaign

ShishAware underwent a process evaluation by looking at longitudinal usage data at three, six, and nine months. This included page traffic, page views, unique trends, and views. We also performed descriptive analyses for our posts and used the number of “likes” and comments as proxies for popularity. Facebook provided a database of user interaction and hence allowed for more detailed analyses than Twitter or YouTube.

## 3. Results

On Facebook, ShishAware posted 130 status updates over nine months (14.4 posts per month), yielding 214 user “likes” and 70 comments. ShishAware had 520 users subscribed at month three, 672 at month six and 776 users at month nine. The majority of users were from the UK (63.9%), male (54.2%), and predominantly aged between 18 and 24 years (63.2%). Sixty-eight and a half percent of status updates had at least one “like”; 23.1% had at least one comment from users. After using an independent samples *t*-test, users were more likely to comment on “shisha facts” than current affairs items (M = 0.29 (SD = 0.70) versus M = 0.10 (SD = 0.31); *P* < 0.05). Longitudinally, there was a strong, positive correlation between the number of weekly active users (as calculated by Facebook) and our weekly posting rate (*r* = 0.71, *n* = 273, *P* < 0.001) and between our weekly posting rate and the page's weekly subscribing rate (*r* = 0.54, *n* = 273, *P* < 0.001).

On Twitter, ShishAware “tweeted” 373 times, averaging 1.4 “tweets”/day. Our longitudinal “tweeting rate” declined over time, from 2.2 “tweets”/day from months 0–3 to 1.1 “tweets”/day from months 3–6 and then to 0.8 “tweets”/day from months 6–9. ShishAware accumulated 563 followers and mainly “tweeted” about current affairs (73.2%). 8.0% of our “tweets” were “retweeted” and nearly two thirds of these (63.0%) were “tweets” mentioning waterpipe tobacco smoking health effects. Other users interacted with ShishAware 70 times (using the notation “@shishaware”), one of which was from a journalist that interviewed ShishAware in person and broadcast the interview on a Somali satellite channel.

On YouTube, ShishAware's video accumulated 7,041 views in six months, and by nine months it had gained 19,428 views. At nine months it gained 69 “likes,” 67 “dislikes,” and 218 comments (112.2 comments/10,000 views) (not including ShishAware's comments), 188 (86%) of which were from pro-waterpipe tobacco smoking individuals. It was also “favourited” by 28 users. YouTube statistics revealed that 76% of viewers were male, and 41% were aged 18 to 34 years, and viewers were from all world continents.

## 4. Discussion

ShishAware was successful in that it was able to reach its target audience, the young, global online community. Not only was ShishAware able to interact with these users on social media, but its campaign resulted in in-person collaboration with a local government organisation and a media interview. ShishAware was able to discern between the types of users for each medium: Facebook attracted supporters of the campaign, YouTube attracted those against the campaign, and Twitter attracted a more balanced mixture of both as well as larger organisations and waterpipe tobacco smoking businesses.

ShishAware also learnt about the suitability of interaction for each medium: Facebook was more suited to more intermittently posted, short health facts and had more interaction with users, whereas Twitter was more suited to daily, external links and current affairs news. The more users joined the Facebook campaign the more active ShishAware was. ShishAware's YouTube video reached the most users (about 20,000) in nine months with no promotional advertising and produced a rich array of user comments that give insight into future improvements of the campaign.

One of the strengths of this case is the use and linkage of multiple social media to reach a young population that may not be as easily reachable through other media. This serves to reduce inequalities in access to healthcare information. ShishAware was also the first public health initiative to produce a social media campaign focussed solely on waterpipe tobacco smoking. ShishAware showed that, with no funding and resources, it was able to engage with the online community and also with the local community in person. The major limitation of ShishAware is the lack of evidence beyond process data. For example, there are no data on the effects on awareness, knowledge, and attitude of media users, let alone data on starting or quitting waterpipe tobacco smoking. More evaluative methods are needed for social media, including the need to measure the quality of health campaigns.

Social media tools for health promotion and education can be broad and varied, ranging from the mainstream tools mentioned in this paper to others such as message boards, e-Games, widgets, and wikis [16]. Large organisations such as the Center for Disease Control (CDC) have a multitude of Facebook Pages and Twitter accounts, each with a substantial number of subscribers. For example, CDC eHealth is dedicated to the use of social media in health promotion that local public health professionals should be made aware of (http://www.cdc.gov/metrics/reports/).

The public campaign for the 2011 World Aids Day (http://www.worldaidsday.org/) is another example of the use of social media in public health campaigns. The public was encouraged to participate by posting pictures of themselves wearing the symbolic red ribbon onto the World Aids Day Facebook page [17]. This example shows how a sense of public attachment was created through user interaction.

We recommend the following: healthcare organisations aiming at raising awareness about waterpipe tobacco smoking should consider social media as an adjunct for healthcare communication. Social media account creation and maintenance should involve its target audience, to take advantage of interlinking social networks and thus attract its target audience, as in ShishAware's case. Responsibility of social media maintenance should rotate across individuals to avoid burnout and enhance interaction variety—something ShishAware probably suffered from. If funding is available, healthcare organisations should seek advertising their campaigns online and developing high-quality videos to expand their reach. A website should be created to allow posting of larger volumes of text, images, and files as a supportive tool to a campaign. Planning stages of social media development should consider setting up intervals of evaluation to monitor progress.

## 5. Conclusions

Social media campaigns are feasible, can be relatively resource nonintensive, and are likely to be effective in raising health awareness. Future studies should assess the effects of social media campaigns on awareness, knowledge, and attitude of media users and ideally the effects on starting or quitting waterpipe tobacco smoking. Researchers need to develop and explore the research methodology and outcome assessment tools for these new types of public health interventions. Further research is needed on validating health information on social media to ensure health messages are accurate and reliable.
